# Depth of field and visual performance after implantation of a new hydrophobic trifocal intraocular lens

**DOI:** 10.1186/s12886-022-02462-3

**Published:** 2022-05-31

**Authors:** Carlos Palomino-Bautista, Alejandro Cerviño, Ricardo Cuiña-Sardiña, David Carmona-Gonzalez, Alfredo Castillo-Gomez, Ruben Sanchez-Jean

**Affiliations:** 1 Department of Ophthalmology, University Hospital Quirón, Madrid, Spain; 2 Department of Optics & Optometry & Vision Sciences, University of Valencia, C / Dr. Moliner, 50, 46100 Burjassot, Valencia Spain

**Keywords:** Depth of field, Trifocal intraocular lens, Defocus curve, Visual performance, Satisfaction

## Abstract

**Purpose:**

To assess the depth of field (DOF) by means of defocus curve analysis applying different visual acuity criteria in patients following cataract surgery and bilateral implantation of a new trifocal diffractive intraocular lens (IOL).

**Methods:**

Fifty eyes of 25 consecutive patients who underwent implantation of the Asqelio™ trifocal IOL (AST Products Inc., USA) were enrolled in this observational prospective study. Monocular subjective DOF was obtained from defocus curves with absolute and relative criteria of tolerance for different visual acuities values. Patient’s visual satisfaction, postoperative refraction and visual acuity at far, intermediate (67 cm) and near (40 cm) distances were also measured at 1 and 3-months post-surgery. Analysis of variance was used to assess differences in refractive error after the surgical procedure, and paired t-tests were used to assess differences in VA. Patient satisfaction results were reported as percentages.

**Results:**

Spherical equivalent was 0.05 ± 0.23 D and residual cylinder 0.01 ± 0.23 D 3-months after the surgery. Absolute DOF obtained was 3.29 ± 0.91 D considering 0.1 LogMAR as cut-off value, and 4.82 ± 0.69 D when 0.3 logMAR as cutoff value. Relative DOF considering a drop of 0.1 logMAR from maximum visual acuity was 2.57 ± 0.82 D, and 1.27 ± 0.70 D when a drop of 0.04 logMAR was considered. Visual acuities obtained 3-months after the surgery were 0.03 ± 0.13, − 0.05 ± 0.06, 0.03 ± 0.08 and 0.04 ± 0.08 logMAR for uncorrected and best-corrected for distance, and best distance-corrected for intermediate and near distances, respectively. Average response to visual satisfaction queries was 8.24/10 at distance, 8.04/10 at intermediate, and 7.88/10 at near.

**Conclusions:**

Patients implanted with this trifocal IOL showed a significant improvement in visual acuity at different distances providing wide absolute and relative DOF values. The outcomes demonstrate that this lens is predictable yielding good patient satisfaction rates.

## Introduction

Trifocal intraocular lens (IOL) implantation in patients submitted to cataract or clear lens surgery has improved the visual outcomes at different distances, from far to near, expanding the range of useful vision and allowing spectacle independence. The benefit of using these lenses in terms of visual performance has been pointed out in several systematic reviews and meta-analysis, where the improvement in visual acuity at intermediate distance is considerable better than that found in patients implanted with bifocal lenses [1–3]. Given the increasing amount of time that people over the age of 45 spend on activities that require intermediate vision, such as computer work or cooking, there is an increasing number of patients implanted with these lenses around the world who improve intermediate vision while maintaining distance vision.

The determination of through-focus visual acuity at different distances (defocus curve) is an increasingly common clinical procedure that proved to be very useful for the assessment of the clinical performance of an IOL at different vergences, particularly those designs aimed to compensate presbyopia (i.e. bifocal, trifocal or enhanced depth of focus IOLs) [4–10]. Among the metrics that can be extracted from the data contained within the defocus curve, the depth of field (DOF) that the lens implanted provides to the patient is one that better allows characterizing the clinical behavior of the IOL. DOF refers to the range of distances (vergences from the defocus curve) where a stimulus can be seen without a significant degradation of the retinal image. Hence, large DOF values correlate with best visual performance at different distances. However, it is important to define the visual acuity criterion being considered as cutoff value. Previous recent reports used 0.1 logMAR as the threshold to define the defocus tolerance [8, 10], however, other values of visual acuity may be used and hence different DOF values can be obtained in the peer-review literature of presbyopia-correcting IOLs. Other authors used a less restrictive criterion to define absolute DOF, suggesting visual acuity values ≤0.3 logMAR [11] since that value is considered as nominally an adequate standard of distance vision for driving, or a more restrictive relative criterion considering a decay of 0.04 logMAR from the best visual acuity as the landmark for individual tolerance [4].

Thus, the main purpose of the present study was to assess the clinical performance of a new single-piece soft hydrophobic trifocal IOL, analyzing the DOF by means of defocus curve analysis using different visual acuity criteria. In addition, postoperative refraction, visual acuity outcomes at different distances, and patient satisfaction were also determined.

## Methods

This was a prospective study involving patients being bilaterally implanted with a trifocal IOL (Asqelio™ Trifocal, AST Products Inc., Billerica, MA, USA). These IOLs were implanted following cataract or clear lens extraction with phacoemulsification and targeted for emmetropia. Inclusion criteria for enrollment included 40 years of age or more, no active ocular disease except for cataract, non-severe dry eye, potential post-operatory best corrected distance visual acuity (CDVA) 0.66 decimal (0.17 logMAR) or better, uneventful cataract surgery and postoperative healing process, clear posterior capsule and lens implant, no pupillary abnormality, capacity to read and understand informed consent and satisfaction questionnaires. Exclusion criteria were irregular corneal astigmatism, previous corneal or intraocular refractive surgery, corneal anomalies, IOL dislocation, posterior capsule opacification, or any vitreous or retinal disease.

Participants were recruited from the Hospital Universitario Quirón (Madrid, Spain), were instructed on the purpose of the study and procedures used and signed a consent form before formal enrollment. In agreement with the Declaration of Helsinki, the protocol of the study was reviewed and approved by the Institutional Ethics Committee.

### Intraocular lens

All patients were implanted with the Asqelio™ Trifocal (AST Products Inc., Billerica, MA, USA) TFLIO130C model IOL. This IOL has a bi-aspheric geometry, with a posterior diffractive optic design (15 rings within the central 4.5 mm) in its 6.0 mm in diameter optical zone. It has a total diameter of 13.0 mm and provides an addition of + 3.30 diopters (D) for near and + 2.20 D for intermediate distances. The lens is built in powers ranging from + 5.00 to + 34.00D in 0.50 D increments, C-Loop platform and with a light distribution among its foci of 50% for distance, 24% for intermediate and 26% for near. It is made of SOF hydrophobic acrylic material (glistening free) with a refractive index of 1.50, Abbe number of 50, and spherical aberration of − 0.27 μm.

### Clinical procedures

Prior to surgery, patients underwent a comprehensive ophthalmological examination including optical biometry and anterior surface optical tomography for the calculation of the power of the IOL. Biometry was determined using the IOLMaster 700 (Carl Zeiss Meditec AG, Germany). The device uses swept source optical coherence tomography technology, with a wavelength of 1050 nm, to determine axial length, anterior chamber depth, and both corneal and lens thicknesses. Additionally, the system provides keratometric readings by analyzing corneal morphology.

IOL calculation was carried out using the biometric parameters provided by the IOLMaster and applying the Barrett II Universal formula. In all cases the IOL power chosen was the one yielding the myopic values closer to zero. No restrictions were applied with regards to axial length. On the contrary, only those patients needing an IOL power within the range + 13 to + 28 D were included.

### Surgical procedure

Surgical procedures were conducted by the same experienced surgeon (CP) under local anesthesia through a micro-incision of 2.2 mm. Surgical procedures with IOL implantation were conducted with a difference of 7 days in average between eyes. All patients were submitted to cataract surgery by phacoemulsification according to regular clinical practice procedures. Surgery was carried out by the same experienced surgeon in all cases (CPB). Although the presence of surgical complications was considered within the exclusion criteria, no surgical complications were observed in the sample recruited.

### Post-operatory assessment

Patients were examined after IOL implantation according to the usual postoperative follow-up visits. During these visits, the visual and ocular status of the patient are revised to rule out the existence of medical complications or IOL-related surprises that might affect the outcomes. Visual acuity was determined with and without best compensation for distance (UDVA and CDVA). Post-operatively, measurements were carried out UCDVA, CDVA, and best distance-corrected intermediate (CDIVA, 67 cm) and near (CDNVA, 40 cm) distances using retroilluminated ETDRS vision chart for distance with Sloan letters (Precision Vision, La Salle, IL) at 85 cd/m^2^, and a Rosebaum chart for visual acuity determination at intermediate and near distances.

Defocus curves were obtained monocularly from all participants under photopic conditions at 3 months post-operatively, following the methodology described by the authors in previous reports [10]. The step size in diopters was 0.50 D, ranging from + 3.00 to − 5.00 D. VA was measured in logMAR scale, and the optotype used was ETDRS (Precision Vision, Illinois, USA) at 4 m. All participants were measured with the best correction for distance to compensate any residual refractive error.

Subjective tolerance to defocus was determined from the analysis of defocus curves, using both absolute and relative criteria. Absolute criterion was obtained from those vergences (in D) which provided visual acuity values ≤0.1 logMAR and relative criterion was obtained considering those vergences (in D) which provided a decay of 0.1 logMAR from the best visual acuity of each subject, as used by the authors in previous studies [8, 10]. Additionally, a second less restrictive criterion was applied to determine absolute DOF, considering those vergences that provided visual acuity values ≤0.3 logMAR [11], and the more restrictive relative criterion considering those vergences (in D) which provided a decay of 0.04 logMAR from the best visual acuity of each subject [4].

### Satisfaction

Patients were asked to grade their level of satisfaction on a scale from 0 to 10, being 10 completely satisfied. Specifically, they were asked to grade their satisfaction with their overall binocular vision, their distance vision, their intermediate vision and their near vision. Additionally, they were asked to score the level of perception and bothersome of halos after IOL implantation on a scale from 0 to 10, being 0 not perceiving/bothered by halos, and 10 very concerned about halos.

### Statistical analysis

Statistical analysis of the results was carried out using IBM® SPSS® for Mac v.26.0.0 (SPSS Inc., Chicago, IL). Analysis of variance was used to assess differences in refractive error after the surgical procedure. Paired samples t-tests were used to assess differences in VA. All statistical tests were 2-tailed, and *p*-values lower than 0.05 were considered as statistically significant. As the satisfaction questionnaires were not validated, responses were not included in the statistical analysis, but the results were reported as percentages.

## Results

A total of 25 cataract surgery patients to be binocularly implanted with the Asqelio Trifocal IOL were enrolled in the study (15 female and 10 male). Descriptive statistics of the sample are displayed in Table 1.

**Table 1:** Descriptive statistics of the sample

	Mean	SD	Range
**Age (ys)**	57	7	46 to 71
**PreOp Sphere (D)**	0,29	1.72	+ 2.75 to −5.25
**PreOp Cylinder (D)**	−0.59	0.48	0 to −1.5
**Flat K (D)**	43.46	1.38	40.61 to 46.95
**Steep K (D)**	44.18	1.37	41.13 to 47.67
**ACD (mm)**	3.08	0.34	2.29 to 4.27
**AL (mm)**	23.43	1.03	22.04 to 25.8
**IOL Power (D)**	21.47	2.65	14 to 25

### Postoperative refraction

**Fig. 1:**
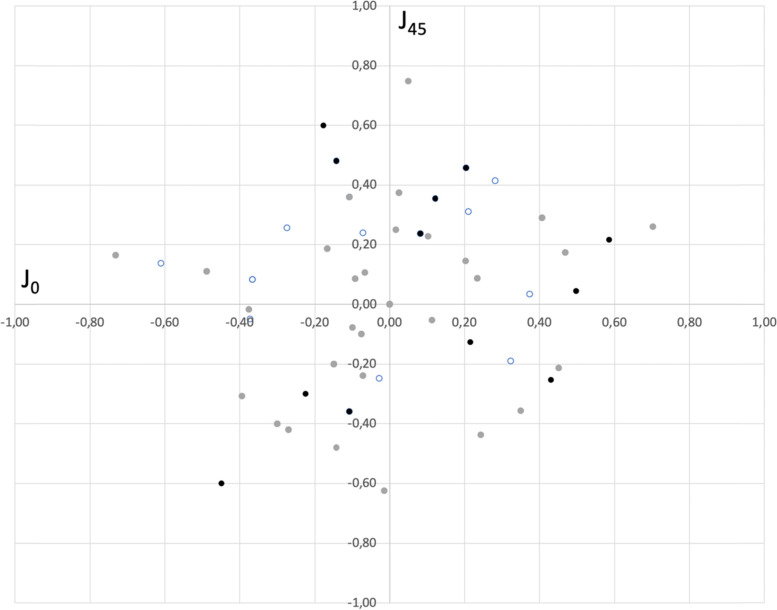
Distribution of residual astigmatism scatterplot (J_0_ and J_45_, diopters). Grey bins represent preoperative data, empty bins 1 month postop, and full black bins 3 months postop

Average spherical equivalent was 0.08 ± 0.34 D at 1 month (range − 0.50 to + 0.75D) and 0.05 ± 0.23 D at 3 months (range − 0.50 to + 0.75D) postoperative. Average residual cylinder was − 0.44 ± 0.41 D at 1 month (range 0 to − 1.25D) and − 0.38 ± 0.48 D at 3 months (range 0 to − 1.5) after the surgery. Figure 1 displays the distribution of residual astigmatism. Analysis of variance of the astigmatic components J_0_ and J_45_ did not reveal statistically significant differences preoperatively and postoperatively (*p* > 0.05).

### Visual acuity

Table 2 displays average visual acuities obtained for far, intermediate, and near distances preoperatively, 1 month and 3 months after surgery. Differences between preoperative uncorrected visual acuity and 3 months postoperative visual acuity were statistically significant (mean difference 0.28 ± 0.25 LogMAR units, *p* < 0.001), almost 3 lines, as well as between DCVA before and 3 months after surgery (mean difference 0.18 ± 0.16 LogMAR units, p < 0.001), almost 2 lines. Differences in visual acuity were not significantly different between 1 month and 3 months postoperatively (*p* > 0.05).

**Table 2:** Average visual acuities obtained for far, intermediate, and near distances preoperatively, one month and three months after surgery (PostOp). (UDVA: uncorrected distance visual acuity; CDVA: best-corrected distance visual acuity; CDIVA: best distance-corrected intermediate visual acuity; CDNVA: best distance-corrected near visual acuity)

Visual acuity (LogMAR)	Mean	SD	Range
**Preoperative UDVA**	0.30	0.24	0.00 to 1.00
**Preoperative CDVA**	0.13	0.16	−0.10 to 0.80
**1 month PostOp UDVA**	0.04	0.12	−0.20 to 0.40
**1 month PostOp CDVA**	−0.04	0.07	−0.20 to 0.14
**3 month PostOp UDVA**	0.03	0.13	−0.18 to 0.44
**3 month PostOp CDVA**	−0.05	0.06	−0.18 to 0.20
**3 month PostOp CDIVA**	0.03	0.08	−0.10 to 0.24
**3 month PostOp CDNVA**	0.04	0.08	−0.14 to 0.30

Given that 29 eyes from the sample were submitted to clear lens extraction, a separate analysis was carried out to determine the difference between CDVA preoperatively (mean value 0.04 ± 0.08 LogMAR) and 3 months postoperatively (− 0.07 ± 0.04 LogMAR), finding statistically significant differences (*p* > 0.001).

Comparing the best distance-corrected visual outcomes between eyes submitted to clear lens extraction (29) and eyes submitted to cataract (21) 3 months after surgery, differences were significant for CDVA (*p* = 0.027) and 3 month PostOp CDNVA (0.004), but less than one line in any case (mean differences were 0.04 ± 0.02 and 0.06 ± 0.02 LogMAR units, respectively).

### Defocus curve

**Fig. 2:**
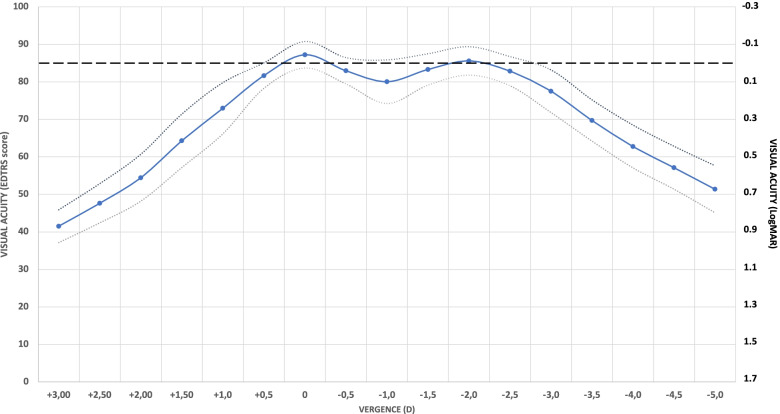
Average defocus curve 3 months after intraocular lens implantation. Dotted curves represent the ±standard deviation curves, and dashed line represents the 0.0 LogMAR

Figure 2 shows the average monocular defocus curve for the whole sample 3 months after IOL implantation. The average visual performance across the defocus curve does not go below 0.1 LogMAR at any point from + 0.50 D to − 2.75 D, yielding an average absolute DOF of 3.35 ± 0.80 D (range 1.00 to 4.50D). The average relative DOF, that is, considering a drop of 0.1 LogMAR from the best visual acuity values, was 2.57 ± 0.82 D (range 0.50 to 5.00D). Considering the alternative criteria described in the methodology, that is, the less restrictive 0.3 logMAR as cut-off visual acuity value for absolute DOF, and a drop of 0.04 logMAR from the maximum visual acuity as a more restrictive criterion for relative DOF, the average values obtained are 4.82 ± 0.69 D (range 3.50 to 6.50D) and 1.27 ± 0.70 D (range 0.00 to 3.50D), respectively.

### Satisfaction

**Fig. 3:**
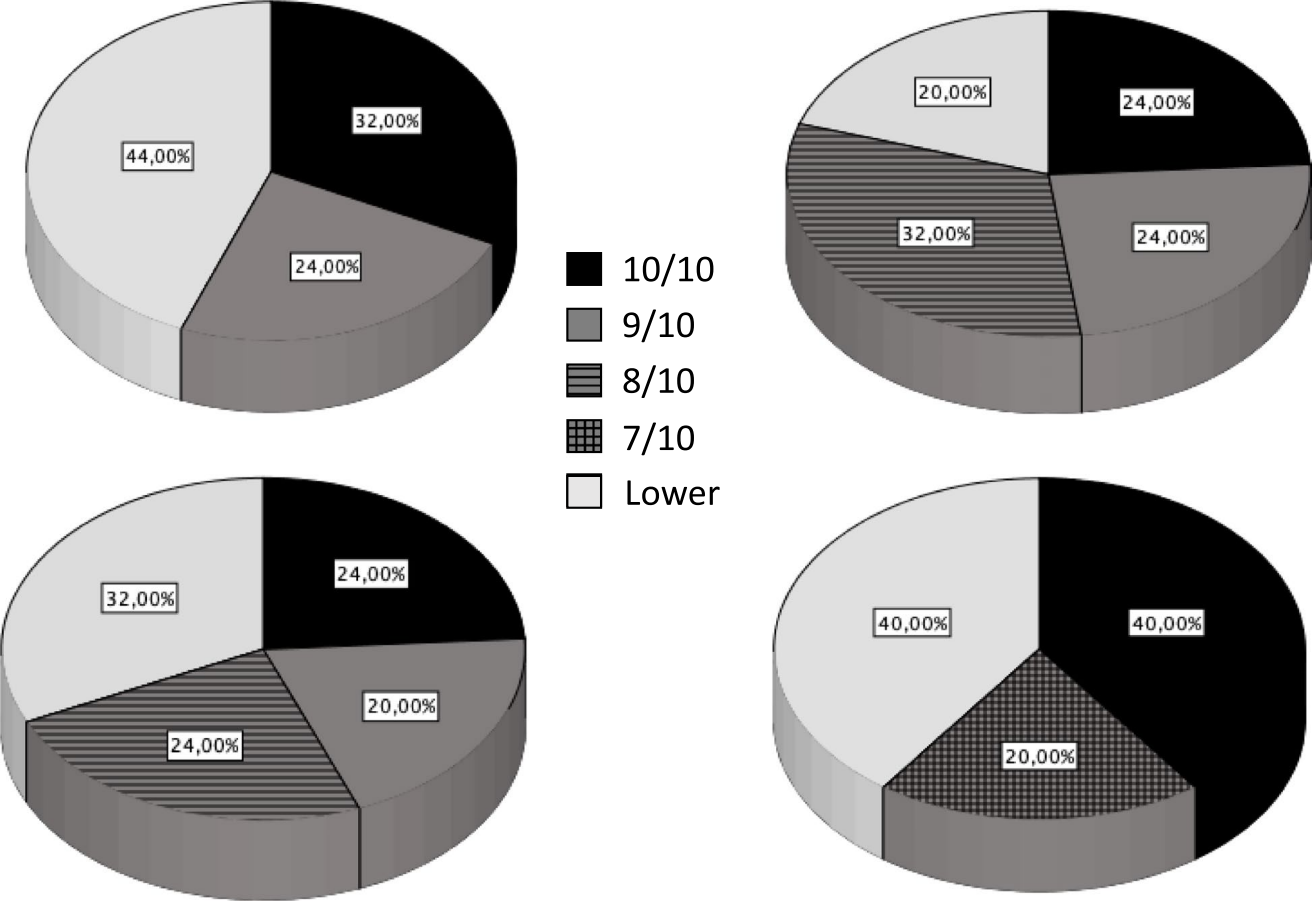
Pie charts showing the distribution of satisfaction scores with regards to vision. Scores with a prevalence lower than 20% were grouped. Overall binocular vision (upper left), Vision for distance (upper right), vision at intermediate distances (lower left), vision at near (lower right)

Average response to satisfaction queries was 8.32/10 for overall satisfaction with the binocular vision, 8.24/10 for visual satisfaction at distance, 8.04/10 for visual satisfaction at intermediate distances, and 7.88/10 for near distances. When asked to score bothersome with regards to halos, being 10 very bothersome and 0 no concern at all, average response was 4.36. Figure 3 displays pie charts showing the distribution of satisfaction scores with regards to vision. 72% of the subjects scored 8/10 or higher with regards to binocular satisfaction, and only 1 subject scored below 5/10. Similarly, 80% scored 8/10 or higher with regards to distance vision, 68% with regards to intermediate vision, and 56% with regards to near vision.

## Discussion

The main purpose of the current study was to determine DOF by means of defocus curve analysis applying different visual acuity criteria in patients following cataract surgery with Asqelio™ trifocal IOL implantation. In addition, standard metrics of postoperative refraction, visual acuity measurement at different distances and patient’s satisfaction were also reported.

In relation to vision and refraction, the outcomes found in the present series of patients indicate that this lens offers good visual acuity from far to near distances and excellent refractive outcomes. A mean spherical equivalent lower than a quarter of diopter was found (0.08D at 1 month and 0.05D at 3 months after the surgery), and mean postoperative cylinder also lower than a quarter of diopter (− 0.03D and 0.01D at 1 and 3 months after the surgery, respectively). Figure 1 shows the power vector of the postoperative astigmatism as depicted by the 2-dimensional vector (J_0_ and J_45_). It should be considered that the [0,0] point represents an eye without astigmatism (center of the graph). The analysis of variance of these astigmatism components did not reveal statistically significant differences preoperatively and postoperatively (*p* > 0.05). Visual acuity outcomes reveal the good performance provided by this lens when implanted. Note that CDVA at 1- and 3-months post-surgery were larger than 20/20 (− 0.04 and − 0.05 logMAR, respectively, see Table 2). At near (40 cm), these values were 0.04 (about 20/22), and at intermediate distance (67 cm) to 0.03 logMAR (about 20/21). There was a statistically significant improvement after surgery (about 3 lines of UDVA at 3 months, *p* < 0.001). During the 3 months of follow-up, visual acuity did not change significantly (*p* > 0.05).

The assessment of the DOF was carried out by means of analysis of the monocular defocus curve, shown in Fig. 3. This curve, at 3-months post-surgery, shows a peak of maximum visual acuity at 0 D vergence (distance vision, > 20/20) and a smooth transition between 0 and − 2.00D (50 cm) vergence. It must be noted that the mean visual acuity performance across the curve does not go below 0.1 logMAR at any point from + 0.50 D to − 2.75 D (about 35 cm), supporting the good visual performance obtained. In the analysis, an average absolute DOF (considering 0.1 LogMAR as cutoff value) of 3.29 ± 0.91 D was found, while the average relative DOF (considering a drop of 0.1 LogMAR) was 2.57 ± 0.82 D. However, taking into account the alternative criteria (0.3 logMAR as cut-off visual acuity value for absolute DOF and a drop of 0.04 for relative DOF values), the average values obtained were 4.82 ± 0.69 D and 1.27 ± 0.70 D, respectively, showing how different results can be obtained depending on the criterion used for defining the cutoff values. The analysis of both relative and absolute criteria is necessary to obtain a complete overview of the any presbyopia-correcting IOL performance [10]. In both cases, the results here obtained reveal the good outcomes reported by these patients. It must be noted though that the defocus curves and DOF reported in the present study are monocular, and therefore an improvement would be expected in binocular metrics, due to binocular summation.

It becomes difficult to compare these results with those from other peer-review studies that analyzed the DOF of several presbyopia-correcting IOLs due to the different DOF criteria used in each study. In a previous study carried out by the authors, DOF after implantation of different bifocal, trifocal and extended depth of focus IOLs was analyzed using the 0.1 LogMAR criteria, as used in the present study [8, 10]. Comparing those outcomes with those here reported, it can be observed that the DOF values obtained in the present study fall within the upper end of the range of DOF values obtained by the IOLs analyzed in the previous studies, and less variability among patients, making the IOL implanted in the present study comparable to those obtaining the best DOF values. The study carried out by Barisic et al. [12], who studied the subjective DOF of the AT LISA TRI IOL, found a mean value of 2.59 D. Note that these authors used a relative criterion of loss of visibility with a letter visual acuity of 20/30. Buckhurst et al. [13], who used an absolute criterion of visual acuity over 0.3 LogMAR, found a DOF about 3 D for the bifocal ReZoom and Tecnics ZM900 IOLs. The DOF values found reported in the present study exceed considerably those reported in those two studies but, as previously indicated, differences between studies might arise, at least in part, from the visual acuity criterion stablished in each study. It has been reported that there is a direct correlation between visual acuity values and DOF (i.e. the higher the visual acuity, the lower the DOF) [14]. There are other potential sources of differences between studies to be considered, such as the time post-surgery when measurements took place, as it may play a role since neuroadaptation in presbyopia-correcting IOL allows the visual system to adapt to the images created on the retina [15], or the step size used for the determination of the defocus curve, such as 0.5 D steps as in the present study compared to studies using 0.25 D steps [16].

Objective DOF values using wavefront aberrometry have been also suggested in comparison to the standard subjective DOF. Analogously to the visual acuity criterion used for subjective DOF determination, an acceptable image quality degradation criterion is used to determine the objective DOF value. Previous studies analyzed and proposed different levels of image quality degradation (from 50 to 90%) to determine objective DOF [8, 10, 17, 18], and therefore different outcomes are reported. As a rule of thumb, a more permissive criterion in retinal image quality degradation provides a higher tolerance and therefore a wider DOF. Objective and subjective measures of DOF have been found not to be comparable due to the wide differences in methodologies and criteria to define the level of degradation acceptance in both visual acuity and optical image quality [10].

With regards to patient’s satisfaction with vision postoperatively, even though a non-validated questionnaire consisting of five questions was used in the present study, the outcomes were included as they may constitute a useful reference for patient satisfaction after surgery. Patients had to score from 1 to 10 their level of satisfaction to the questions related to their overall binocular distance vision, their vision at distance, their vision at intermediate distances, and their vision for near distances. The fifth question was related to the incidence and bothersome of halos after IOL implantation, being 0 the highest satisfaction level. The analysis of responses shows that the overall level of satisfaction is high, with more than half of the patients scoring 8/10 or higher their level of satisfaction at each of the distances, while only two subjects scored 8/10 or higher their level of bothersome with halos. Although these results may serve as a reference of satisfaction levels reported by patients implanted with this IOL, satisfaction metrics would need however further study using specific validated questionnaires and appropriate sample sizes to allow appropriate conclusions to be drawn.

In conclusion, patients submitted to phacoemulsification for cataract surgery and implanted with this new trifocal IOL showed a significant improvement in visual performance at different distances. These preliminary outcomes show the lens as predictable, providing wide absolute and relative DOF values, allowing good visual acuity at different distances and good patient satisfaction rates.

## Data Availability

The datasets used and/or analysed during the current study are available from the corresponding author on reasonable request.
